# 541. Global Clinical Evidence Demonstrates Skin Cell Suspension Autograft as a Standard of Care for Wounds

**DOI:** 10.1093/jbcr/irag033.238

**Published:** 2026-04-06

**Authors:** C Scott Hultman, Anju Saraswat, James H Holmes IV

**Affiliations:** WakeMed Health and Hospitals, Raleigh, NC; Atrium Health Wake Forest Baptist, Winston-Salem, NC; Atrium Health Wake Forest Baptist Burn Center, Winston-Salem, NC

## Abstract

**Introduction:**

Skin cell suspension autograft (SCSA) enables robust closure while significantly minimizing the burden of the trauma. Generated from a small sample of the patient’s own skin, SCSA provides expansion ratios of up to 1:80, reducing donor site size, pain, healing time, and scarring. Although this approach has been increasingly adopted, the available clinical evidence across wound etiologies, depths, patient populations, and study designs has not been comprehensively synthesized. This systematic review was conducted to characterize published evidence on the use of SCSA for wound management to better inform clinical practice and guide future research.

**Methods:**

A systematic search of PubMed and Google Scholar databases were performed for clinical studies published between January 2000 and December 2024. Studies were included if they reported on the clinical use of SCSA for wound closure. Extracted data included information on study design, patient demographics, wound characteristics, and treatment details. Comparative studies were additionally assessed for efficacy endpoints.

**Results:**

Ninety-nine studies were included, representing more than 8000 pediatric and adult patients receiving either SCSA or other treatment, 13 countries, and all levels of evidence. Wound types included burns, surgical wounds, traumatic injuries, inflammatory conditions, and non-healing wounds of multiple depths. Reported wound sizes ranged from 1 cm^2^ to >90% TBSA. More than 30 adjunctive modalities were reported, including enzymatic and hydrosurgical debridement, allograft, xenograft, dermal matrices, and advanced dressings. Trends identified in comparative studies (n = 27) included SCSA association with reduced donor site morbidity, faster healing, similar or improved functional/aesthetic outcomes, and reductions in hospital stay and resource use.

**Conclusions:**

The global body of clinical evidence demonstrates SCSA has been used across diverse patient populations and wound types. Treatment achieves wound closure while minimizing donor site morbidity and hospitalization, and its compatibility with existing surgical workflows supports broad clinical adoption.

**Applicability of Research to Practice:**

Findings from this review reinforce the role of SCSA as a standard component of wound management. Its clinical advantages translate into meaningful improvements for patients, while reductions in resource utilization and overall healthcare costs highlight value for hospitals and health systems.

**Funding for the study:**

This study was funded by the manufacturer.

